# Proteomic analysis reveals resistance mechanism against biofuel hexane in *Synechocystis* sp. PCC 6803

**DOI:** 10.1186/1754-6834-5-68

**Published:** 2012-09-07

**Authors:** Jie Liu, Lei Chen, Jiangxin Wang, Jianjun Qiao, Weiwen Zhang

**Affiliations:** 1 School of Chemical Engineering & Technology, Tianjin University, Tianjin 300072, P.R. China; 2 Key Laboratory of Systems Bioengineering, Ministry of Education of China, Tianjin 300072, P.R. China

**Keywords:** Hexane, Tolerance, Proteomics, *Synechocystis*

## Abstract

**Background:**

Recent studies have demonstrated that photosynthetic cyanobacteria could be an excellent cell factory to produce renewable biofuels and chemicals due to their capability to utilize solar energy and CO_2_ as the sole energy and carbon sources. Biosynthesis of carbon-neutral biofuel alkanes with good chemical and physical properties has been proposed. However, to make the process economically feasible, one major hurdle to improve the low cell tolerance to alkanes needed to be overcome.

**Results:**

Towards the goal to develop robust and high-alkane-tolerant hosts, in this study, the responses of model cyanobacterial *Synechocystis* PCC 6803 to hexane, a representative of alkane, were investigated using a quantitative proteomics approach with iTRAQ - LC-MS/MS technologies. In total, 1,492 unique proteins were identified, representing about 42% of all predicted protein in the *Synechocystis* genome. Among all proteins identified, a total of 164 and 77 proteins were found up- and down-regulated, respectively. Functional annotation and KEGG pathway enrichment analyses showed that common stress responses were induced by hexane in *Synechocystis*. Notably, a large number of transporters and membrane-bound proteins, proteins against oxidative stress and proteins related to sulfur relay system and photosynthesis were induced, suggesting that they are possibly the major protection mechanisms against hexane toxicity.

**Conclusion:**

The study provided the first comprehensive view of the complicated molecular mechanism employed by cyanobacterial model species, *Synechocystis* to defend against hexane stress. The study also provided a list of potential targets to engineer *Synechocystis* against hexane stress.

## Background

Human society has been depending on fossil fuels in the past centuries. However, fossil fuels are not infinite resources, and a sharp price increase of these natural resources in recent years has posed an urgent call for alternative ways to produce fuels and chemicals. Moreover, over-utilizing fossil fuels has also caused environmental issues such as global warming and pollution. To address these issues, photosynthetic cyanobacteria have attracted significant attention recently as a cell factory to produce renewable biofuels and chemicals due to their capability to utilize solar energy and CO_2_ as the sole energy and carbon sources [1-4]. In addition, cyanobacteria have a relatively simple genetic background and are easy for genetic manipulation [5,6]. In recent studies, two approaches have been taken to utilize cyanobacteria for biofuel production: *i*) to isolate fatty acids from lipid-rich cyanobacterial biomass and then convert them chemically to other products, such as biodiesel [7,8]. However, lipid extraction process from cyanobacteria is very energy-intensive and has been one of the major hurdles for commercial application [9-11]; and *ii*) to employ genetically manipulated cyanobacteria to produce secretable fuel products directly. So far the second approach has led to successful production of a dozen of fuel products in engineered cyanobacterial systems, including ethanol [12,13], ethylene [14], isoprene [15], free fatty acids [16], fatty alcohols [17], isobutyraldehyde [18], 1-butanol [19,20] and hydrogen [21,22]. Although the current productivity level by these systems is still very low, the studies clearly demonstrated the feasibility of developing sustainable production systems based on cyanobacterial cells.

Biofuels offer a diverse range of promising alternatives. Although currently ethanol constitutes 90% of all biofuels in the United States, other fuels with better chemical properties, such as bio-based alkane due to their low water solubility and high energy density [23], are also being pursued around the world [24,25]. Alkanes composed of 5 to 9 carbons, which are liquid at room temperature and among the usual suspects in gasoline, can be used as a good fuel in internal combustion engine [26], while C8–C21 alkanes are the predominant components of diesel fuel [27]. Biosynthesis of alkanes has also been reported in a diversity of microorganisms including photosynthetic cyanobacteria since later 1960s [24,27,28]; however, its production in native producing hosts has not received much attention due to their low productivity. In a recent study, the researchers isolated a biosynthesis pathway consisting of an acyl-acyl carrier protein reductase and an aldehyde decarbonylase, which together convert intermediates of fatty acid metabolism to alkanes and alkenes in cyanobacterium *Synechococcus elongatus*, and expressed it heterologously in *Escherichia coli*, leading to the production and secretion of C13 to C17 mixtures of alkanes at ~ 0.3 g/L after 40 h cultivation in *E. coli*[29]. The work for the first time demonstrated the potential to use heterologous hosts for high–efficiency alkane production. Currently efforts are ongoing in both academic and industry settings to express synthetic alkane pathways in photosynthetic cyanobacterial hosts for the production of the third-generation carbon-neutral biofuels.

As solvents, alkane products are toxic to microbes [30]. Their toxicity is inversely correlated with the log*P*_ow_ value, which is the common logarithm of the partition coefficient (*P*_ow_) for the distribution of the organic solvent between *n*-octanol and water phases [31,32]. A series of genes involved in alkane tolerance in *E. coli* have been identified and utilized as targets to improve alkane tolerance by genetic engineering, which has led to some progress in improving alkane tolerance in *E. coli*[33-37]. Cyanobacteria have low tolerance to alkanes; meanwhile, currently the knowledge on alkane tolerance in cyanobacteria is very limiting. To fully understand the effects of alkane on the cyanobacterial cells so that a construction of more robust alkane-producing cyanobacterial hosts can be possible, in this study, we employed a quantitative proteomics approach with isobaric tag for relative and absolute quantification (iTRAQ) technique and liquid chromatography-tandem mass spectrometry (LC-MS/MS) to reveal the global metabolic response of the model cyanobacterium *Synechocystis* to the treatment of hexane, a representative alkane. The results showed that common stress responses which have been reported for other microbes under solvent/biofuel stress were induced by hexane in *Synechocystis*. Notably, the analysis revealed the induction of large numbers of transporters and membrane-bound proteins, proteins related to sulfur relay system, oxidative stress response and photosynthesis, suggesting that they were among the major protection mechanisms against hexane. The study provided the first comprehensive view of the complicated molecular mechanism employed by cyanobacterial model species, *Synechocystis* to defend against hexane stress, and also constituted an important foundation for rational engineering of more robust photosynthetic hosts for the production of the carbon-neutral biofuel alkane.

## Results and discussion

### Hexane effect on *Synechocystis* sp. PCC 6803

**Figure 1:**
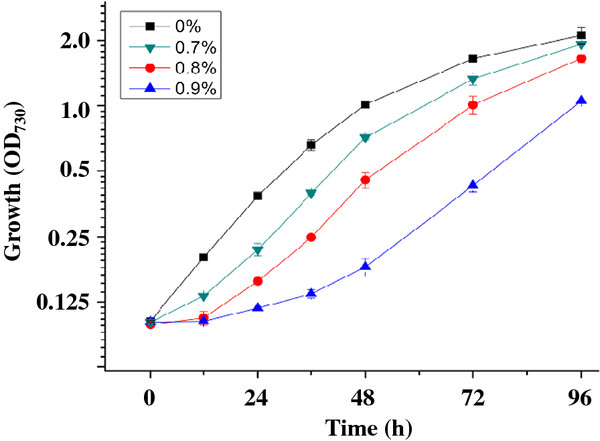
**Effects of hexane. **Growth time courses with varying concentration of hexane.

The growth of *Synechocystis* supplemented with 0, 0.7%, 0.8% and 0.9% hexane was assessed to determine an appropriate hexane concentration for proteomic studies (Figure 1). The results showed that the hexane concentration that caused an approximately 50% growth inhibition was found to be 0.8% (*v*/*v*) at both 24 h and 48 h (corresponding to middle-exponential and exponential-stationary transition phases, respectively), and was selected for the proteomics analysis in this study. The tolerance level of *Synechocystis* to hexane was similar to what has been reported for *Aeromonas hydrophila* and *Pseudomonas aeruginosa*[38]. Cell morphology examination showed no visible change after hexane treatment (data not shown). For proteomic analysis, two independent cultivations for both control and 0.8% hexane treatment were conducted, and cells were collected by centrifugation (8,000 x *g* for 10 min at 4°C) at 24 h and 48 h, resulting in two biological replicates for each time point of control and hexane-treated samples (Figure 1). 

### Overview of quantitative proteomics

**Figure 2:**
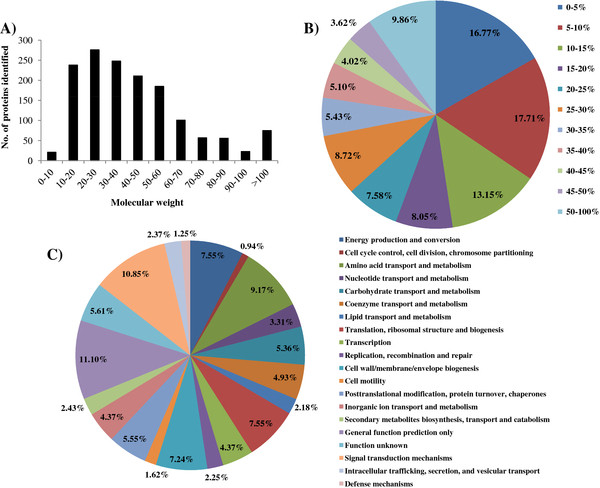
**Distribution, coverage, and functional category of proteins identified in this study. A) **Distribution of protein identified among different molecular weights; **B) **Coverage of proteins by the identified peptides; **C) ** Functional category coverage of the proteins identified.

A total of 167,191 spectra were obtained from the iTRAQ - LC-MS/MS proteomic analysis. After data filtering to eliminate low-scoring spectra, a total of 24,162 unique spectra that met the strict confidence criteria for identification were matched to 1,491 unique proteins, representing approximately 41.8% of the 3569 predicted proteins in the *Synechocystis* genome ( Additional file 1: Table S1). In terms of protein molecular weight (MW) distribution, good coverage (averages of 30–45% of the total proteins in each MW group) was obtained for a wide MW range for proteins larger than 10 kDa (Figure 2A). In addition, most of the proteins were identified with good peptide coverage, of which ~65% of the proteins were with more than 10% of the sequence coverage, and ~44% were with 20% of the sequence coverage (Figure 2B). Among all the proteins detected, 1,181 and 1,172 were identified from the samples of 24 h and 48 h, respectively. The proteins that were identified only in control or treated samples so that ratio calculation is not available were excluded from the analysis. Based on the number of unique proteins identified in each functional category, the most frequently detected functional categories were “general function prediction only” and “signal transduction mechanisms”, representing 11.1% and 10.85% of all the protein identified, respectively (Figure 2C). Proteins involved in signal transduction network are generally with low abundance, quick protein turnover time and membrane-bound [39], a high coverage of the group of signal proteins also suggested the methodology used in the study is with high sensitivity and very reliable. The high percentage of functionally unknown proteins identified is not unreasonable, considering more than 40% of proteins in the *Synechocystis* genome are still annotated as hypothetical proteins without any functional prediction. Other most frequently detected functional categories included “amino acid transportation and metabolism” (9.17%), “energy production and conversion” (7.55%), and “translation, ribosomal structure and biogenesis” (7.55%) and “cell wall/membrane/envelope biogenesis” (7.24%), suggested that the proteins in these functional categories were highly expressed and may be active during the growth and treatment conditions. 

**Figure 3:**
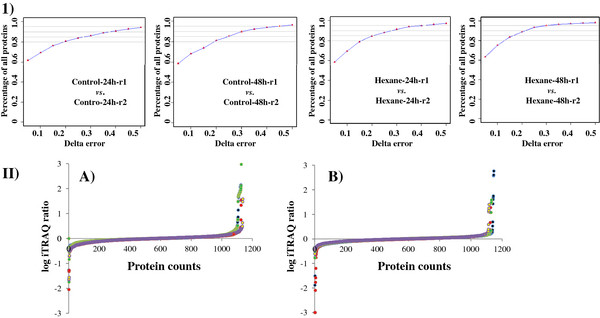
**Reproducibility of proteomic analysis. I**) Reproducibility between biological replicates. Hexane-treated biological replicates at 24 h (**A**) and 48 h (**B**), respectively. **II**) Distribution of iTRAQ log ratios of the 1181 and 1172 proteins identified at 24 h (**A**) and 48 h (**B**) among four biological replicates, respectively. The four sets of biological replicates at 24 h were Hexane-24 h-r1 *vs. *Control-24 h-r1, Hexane-24 h-r2 *vs. *Control-24 h-r1, Hexane-24 h-r1 *vs. *Control-24 h-r2, and Hexane-24 h-r2 *vs. *Control-24 h-r2, indicated by different colors. The four sets of biological replicates at 48 h were Hexane-48 h-r1 *vs. * Control-48 h-r1, Hexane-48 h-r2 *vs. *Control-48 h-r1, Hexane-48 h-r1 *vs. * Control-48 h-r2, and Hexane-48 h-r2 *vs. *Control-48 h-r2, indicated by different colors.

Reproducibility of the proteomic analysis was assessed in two types of comparisons (Figure 3). First we labeled and mixed two biological replicates of a given condition directly for proteomic analysis (*i.e.* biological replicate 1 and 2 of control at 24 h, replicate 1 and 2 of control at 48 h, biological replicate 1 and 2 of hexane treatment at 24 h, biological replicate 1 and 2 of hexane treatment at 48 h), the difference was plotted verse the percentage of the proteins identified, the results showed that approximately 60% of the proteins with difference less than delta error of 0.1, and more than 95% of the proteins with difference less than delta error of 0.5 (Figure 3-I). Second we labeled and mixed each pair of hexane-treated sample and its control for proteomic analysis, the difference between different biological pairs was plotted in Figure 3-II. The dispersion of the iTRAQ ratios of the quantified proteins (*i.e.* 1,181 and 1,172 for 24 h and 48 h, respectively) was found with very similar trends between four biological replicates at either 24 h (Figure 3-II-A) or 48 h (Figure 3-II-B), suggesting that the biological noise was reasonably low.

### Pathway enrichment analysis of the hexane-responsive proteins

Using a cutoff of 1.5-fold change and a *p*-value less than 0.05, we determined that 140 and 148 unique proteins were differentially regulated between control and hexane treatments conditions at 24 h and 48 h, respectively ( Additional file 1: Table S1). Among them, a total of 164 and 77 proteins were up-regulated and down-regulated by the hexane treatments, respectively. Forty-two up-regulated and 4 down-regulated proteins were shared between 24 h and 48 h, while more of the responsive proteins were unique for each of the time points, consistent with the expected physiological differences between middle-exponential and transition phases of the cell growth (Figure 1).

Metabolic pathway enrichment analysis was carried out for the differentially regulated proteins to determine the affected cellular metabolism ( Additional file 2: Table S2). The analysis was performed by matching the responsive proteins to the proteins annotated with KEGG Pathway database, and then comparing the frequencies of the responsive proteins in each KEGG pathway to determine statistically authenticity of the involvement of that KEGG pathway in hexane response. A series of KEGG pathways affected by the hexane treatment, with *p*-value less than 0.05 as cut off were identified. The results showed that at 24 h after hexane treatment, four KEGG pathways were differentially regulated by the hexane treatment: they are “Ribosome” (KO03010), “Sulfur relay system” (KO04122), “Photosynthesis” (KO00195) and “Arachidonic acid metabolism” (KO00590). All four pathways were enriched by at least two out of four biological replicates, with “Ribosome” (KO03010) enriched with good confidence in all four replicates and “Photosynthesis” (KO00195) enriched in two replicates, consistent with the relatively high expression of ribosomal proteins and photosynthetic proteins, and the sensitivity of protein biosynthesis and photosynthetic processes to stress. Most of the ribosomal proteins identified were down-regulated, suggesting an overall slowdown of the protein biosynthesis and possible slow metabolism. At 48 h, five KGG pathways, “Sulfur relay system” (KO04122), “ABC transporters” (KO02010), “Photosynthesis” (KO00195), “Steroid biosynthesis” (KO00100), and “Biosynthesis of ansamycins” (KO01051) were enriched by the differentially expressed proteins. Although these KEGG pathways were enriched in only two out of four biological replicates, the *p*-values were reasonably low, suggesting in general the reliability of the data analysis. The enrichment of “Ribosome” pathway (KO03010) at only 24 h was interesting, which may implicate that modification of the primary metabolism, such as protein biosynthesis, may be one of the major strategies that cells used to deal with stress during middle-exponential phase. In this study, we found that several ribosomal proteins, Sll1816 (*rpsM*), Sll1803 (*rplV*) and Ssr0482 (*rpsP*) were down-regulated under hexane treatment. Interestingly, these three genes, *rpsM*, *rplV*, and *rpsP*, were named as targets as they were also down-regulated in *E. coli* against organic solvent also indicated [35].

“Sulfur relay system” (KO04122) was differentially regulated at both 24 and 48 h, in three out of four biological replicates at 24 h, and two out of four biological replicates at 48 h, respectively ( Additional file 2: Table S2). The sulfur-relay system classified in tRNA modification has been demonstrated to modify a few nucleotides of tRNA molecules, their increased expression has been reported contributing to stabilization of their structure, and was required for survival at an extremely high temperature in *E. coli*[40] and *Thermous thermophilus*[41]. In addition, ubiquitin (Ub) like proteins are signaling messengers that control many cellular functions in bacteria. It has been proposed that the Ub-protein modification evolved from prokaryotic sulfurtransfer systems [42]. Molybdenum cofactor (Moco) and thiamin are sulfur-containing cofactors whose biosynthesis includes a key sulfur transfer step that uses unique sulfur carrier proteins, MoaD and ThiS. Detailed analysis showed that upon hexane stress, two proteins in “Sulfur relay system” pathway, Slr0821 and Ssl1707 with homologies to sulfur carrier proteins MoaD and sulfur-accepting protein TusA were up-regulated, consistent with their possible roles against stress.

As one core function of cyanobacteria, “Photosynthesis” pathway (KO00195) was differentially regulated by hexane exposure at both 24 h and 48 h, in two out of four biological replicates ( Additional file 2: Table S2). Early studies showed that some natural stresses, such as salt and sulfur starvation, decreased expression level of genes for phycobilisome, photosystems I and II, cytochrome b6/f, and ATP synthase, indicating overall reduced light-harvesting and photosynthetic activity upon stress [43,44]. However, detailed analysis showed that six proteins involved in photosystem I and II (Slr0172: photosystem I assembly protein; Sll0629: photosystem I subunit X; Slr1645: photosystem II 11 kD protein; Sll1194: photosystem II complex extrinsic protein precursor U; Sll1418: photosystem II oxygen-evolving complex 23 K protein PsbP homolog; and Sll1398: photosystem II reaction center protein Psb28), four ferredoxin proteins (Slr1828, Ssl0020, Sll1382, Slr1205) and two cytochrome (Ssr3451: cytochrome B559 subunit alpha; and Sll0258: cytochrome C-550) were all up-regulated by hexane exposure (Table 1). Although it remains unclear how the increased expression of photosynthesis-related proteins will help combat the hexane toxicity, the results were consistent with our recent study on ethanol toxicity in *Synechocystis* sp. PCC 6803, where the similar proteomics analysis also demonstrated a profoundly increased expression of many proteins related to photosynthesis [45]. 

**Table 1:** Up-regulated proteins by hexane *,**,***

**Protein ID**	**24 h**				**48 h**				**Description**
	**Hexane-24h-r1 vs Control-24h-r1**	**Hexane-24h-r2 vs Control-24h-r1**	**Hexane-24h-r1 vs Control-24h-r2**	**Hexane-24h-r2 vs Control-24h-r2**	**Hexane-48h-r1 vs Control-48h-r1**	**Hexane-48h-r2 vs Control-48h-r1**	**Hexane-48h-r1 vs Control-48h-r2**	**Hexane-48h-r2 vs Control-48h-r2**	
Slr0250								1.74	17.3 kd protein mura rpon intergenic region precursor
Sll1804							1.55		30S ribo somal protein S3
Slr0444							1.54		3-phosphoshikimate 1-carboxyvinyltransterase
Sll1821						1.80			50S ribosomal protein L13
Sll1813					1.60				50S ribosomal protein L15
Ssl3436			2.58	2.06	1.72	1.71			50S ribosomal protein L29
Ssl1426								2.07	50S ribosomal protein L35
Ssl2084	3.11	3.46	3.55	5.13	1.95				Acyl carrier protein
Sll1017							1.93		Ammonium/methylammonium permease
Slr0242				1.58	1.66				Bacterioferritin co migratory protein
Slr0043							2.56		Bicarbonate transport system ATP-binding protein
Slr0041					3.60				Bicarbonate transport system permease protein
Sll0834	1.54								Bicarbonate transporter
Slr0436							1.66		Carbon dioxide concentrating mechanism protein Ccmk
Slr1853						1.61			Carboxymuconolactone one decarboxylase
Slr2131			1.66	1.89		1.56			Cation or drug efflux system protein
Sll0672							1.63		Cation-transporting ATPase E1-E2 ATPase
Sll1895							1.80	1.71	C-di-GMP phosphodiesterase A
Sll0039			1.83	1.78					CheY family protein positive phototaxis protein
Slr0757			1.79	1.68					Cysteine clock protein KaiB
Sll0712		2.04							Cystteine synthase A
Ssr3451	2.40	2.34	1.66					1.54	Cytochrome B559 subunit alpha
Sll0258						1.55			Cytochrome C-550
Sll1712				1.80					DNA binding protein HU
Sll1699								2.55	Extracellular solute binding
Slr1330						1.52			F0F1 ATP synthase subunit epsilon
Slr1828			1.59	1.52	1.59	1.79		1.63	Ferredoxin
Ssl0020		1.62			1.82	2.04			Ferredoxin
Sll1382					1.61				Ferredoxin
Slr1205			2.02	1.56					Ferredoxin component
Slr1490	2.04	2.13						1.64	Ferrichrome-iron receptor
Slr1761			2.09	2.01	1.51				FKBP-type peptidyl prolyl cis-trans isomerase
Slr1269								1.52	Gamma-glutamyltranspeptidase
Slr0033	1.56		2.45	2.36	1.70	1.79			Glutamyl-tRNA(Gln) amidotransterase subunit C
Slr1992			2.07	2.23	1.68	1.63			Glutathione peroxidase
Slr1171			1.54	1.59					Glutathione peroxidase
Slr0879						1.50			Glycine decarboxylase complex H-protein
Sll0404					1.54	1.79			Glycolate oxidase subunit GlcD
Sll0057			1.94	1.75	1.52	1.59			Heat shock protein GrpE
Slr0298			1.80		1.75				Heterocyst to vegetative cell connection protein
Ssl3044				2.08					Hydrogenase component
Slr0689	1.68					1.59			Inosine-5-monophosphate dehydrogenase related protein
Sll1558							1.59		Mannose-l-phosphate guanyltransterase
Ssr2857						1.51			Mercuric transport protein periplasmic component precursor
Sll1394			1.53						Methionine sulfoxide reductase A
Sll0689	1.85								Na/H^+^ antiporter
Sll0493			1.77						Na-activated K transporter subunit KtrA
Slr0891							1.56	1.51	N-acetylmuramoyl-L-alanine amidase
Sll0223		2.22		2.08					NAD (P) H-quinone oxidoreductioase subunit 2
Sll1262						1.52			NAD (P) H-quinone oxidoreductioase subunit N
Ssl2667			2.92	2.82					NifU protein
Slr0513					1.58	1.81		1.58	Periplasmic iron-binding protein
Slr1160						1.66		1.51	Periplasmic protein, function unknown
Slr2144							3.66		Periplasmic protein, function unknown
Slr1513					4.31				Periplasmic protein, function unknown
Sll0319				1.57					Periplasmic protein, function unknown
Sll0172			1.84	1.56					photosystem l assembly protein
Sll0629		2.86							photosystem l subunit X
Slr1645	1.51	1.64							Phostosystem II 11kD protein
Sll1194						2.88			photosystem ll complex extrinsic protein precursor U
Sll1418							1.51		photosystem ll oxygen-evolving complex 23K protein
Sll1398			1.79	1.62	1.65	1.83			Photosystem ll reaction center protein Psb28
Sll0617				2.01	1.71	1.98			Plasma membrane protein essential for thylakoid formation
Sll0199			1.77			1.61			Plastocyanin
Sll0779	1.83		1.88						PleD-like-protein
Sll0043			1.72						Positive phototaxis protein
Slr0775			1.68						Preprotein translocase subunit SecF
Slr0042					1.56		1.69		Probable porin: major outer membrane protein
Sll1734				2.19	1.98		1.55		Protein involved in low CO_2_-inducible, high affinity CO_2_ uptake
Ssr2049			1.54						Protochlorophillide reductiase 57 kD subunit
Sll0135							2.20	1.93	Putative 5’-methylthioadenosine phosphorylase
Slr0821	1.55	1.59	2.34	2.36	1.74	2.23			Putative sulfur carrier protein slr0821;
Slr1046	1.56								Putative T at A protein
Sll1440						1.59			Pyridoxamine 5’-phosphate oxidase
Sll1277						2.25			Recombination protein F
Slr1198				1.74	1.54	1.52			Rehydrin
Slr1164			3.34						Ribonucleotide reductase subunit alpha
Sll0469								1.51	Ribose-phosphate pyrophosphokinase
Slr0193						1.52			RNA-binding protein
Sll1549					5.32				Salt-enhanced periplasmic protein
Ssl3335							1.71		Secretory protein SecE
Sll0698			1.66						Sensor-like histidine kinase
Sll0288								2.02	Septum formation inhibitor
Sll1284							1.68		Serine esterase
Slr1697								2.26	Serine/threonine kinase
Slr1531								1.68	Signal recognation particle protein
Sll1366					1.76				Snf2/Rad54 family helicase
Slr1512					2.67				Sodium-dependent bicarbonate transporter
Sll1023							1.61		Succinate--CoA ligase
Slr1034			1.74	1.53					Thylakoid-associated single-stranded DNA-binding protein
Sll1742			1.63						Transcription antitermination protein NusG
Sll1483					2.21	2.43			Transforming growth factor induced protein
Slr0709						1.53			Translation linitiation inhibitor
Sll0162, Sll0272, Sll0274, Sll0293, Sll0381, Sll0470, Sll0588, Sll0623, Sll0630, Sll0860, Sll0872, Sll0931, Sll1201, Sll1289, Sll1532, Sll1594, Sll1618, Sll1774, Sll1783, Sll1873, Sll1911, Slr0006, Slr0013, Slr0038, Slr0111, Slr0318, Slr0333, Slr0420, Slr0455, Slr0476, Slr0503, Slr0565, Slr0575, Slr0601, Slr0613, Slr0680, Slr0729, Slr0848, Slr0867, Slr0923, Slr0924, Slr0955, Slr1236, Slr1266, Slr1273, Slr1417, Slr1438, Slr1472, Slr1557, Slr1623, Slr1649, Slr1690, Slr1732, Slr1846, Slr1847, Slr2101, Ssl0242, Ssl0352, Ssl0467, Ssl0832, Ssl1690, Ssl1707, Ssl1972, Ssl2717, Ssl3364, Ssr1528, Ssr1853, Ssr2554, Ssr3122, Ssr3304, Ssr3402									Hypothetical proteins

*Proteins with1.5 fold change and *p - *value less than 0.05.

**Hypothetical proteins listed with gene ID only, full information in Additional file 1: Table S1.

***Blank represents that ratio can’t be calculated for the condition.

“Arachidonic acid metabolism” pathway (KO00590) was differentially regulated by hexane exposure at 24 h, in two out of four biological replicates ( Additional file 2: Table S2). Detailed analysis showed two proteins of glutathione peroxidase (Slr1992 and Slr1171) were up-regulated and thus contributed to the enrichment of this pathway (Table 1). Peroxidases are heme-cofactored enzymes responsible for hydrogen peroxide removal, cytoplasmic glutathione peroxidase (Gpx) has been found involved in oxidative defense in many bacteria [46].

“Steroid biosynthesis” pathway (KO00100) was differentially regulated by hexane exposure at 48 h ( Additional file 2: Table S2). Detailed analysis showed that *sll0513* encoding a putative squalene synthase was down-regulated, which then contributed to the enrichment of this pathway (Table 2). Role of this enzyme in cyanobacteria upon hexane stress still needs more investigation, but it has been reported that squalene synthase (Erg9) in yeast was down regulated upon oxidative stress and ergosterol level plays a key role in adaptation to oxidative stress [47]. 

**Table 2:** Down-regulated proteins by hexane *,**,***

**Protein ID**	**24 h**				**48 h**				**Description**
	**Hexane-24h-r1 vs Control-24h-r1**	**Hexane-24h-r2 vs Control-24h-r1**	**Hexane-24h-r1 vs Control-24h-r2**	**Hexane-24h-r2 vs Control-24h-r2**	**Hexane-48h-r1 vs Control-48h-r1**	**Hexane-48h-r2 vs Control-48h-r1**	**Hexane-48h-r1 vs Control-48h-r2**	**Hexane-48h-r2 vs Control-48h-r2**	
Sll1605					0.60				(3R)-hydroxymyristoyl-ACP dehydratase
Sll1101							0.59	0.58	30S ribosomal protein S10
Sll1816	0.60	0.37	0.64	0.49					30S ribosomal protein S13
Slr0628			0.38	0.35					30S ribosomal protein S14
Ssr0482	0.52	0.48		0.66					30S ribosomal protein S16
Ssl3437	0.49		0.63						30S ribosomal protein S17
Ssr1399		0.52							30S ribosomal protein S18
Sll1804		0.61		0.54					30S ribosomal protein S3
Slr0469	0.47	0.41	0.59	0.47					30S ribosomal protein S4
Sll1812		0.62							30S ribosomal protein S5
Sll1097	0.54								30S ribosomal protein S7
Sll1809	0.57	0.48	0.48	0.45					30S ribosomal protein S8
Sll1822	0.47	0.32	0.63	0.48					30S ribosomal protein S9
Slr1140					0.65	0.65			3-amino-5-hydroxybenzoic acid synthase
Sll1821	0.42	0.35	0.57	0.48			0.65		50S ribosomal protein L13
Sll1813	0.53	0.41	0.64	0.55					50S ribosomal protein L15
Sll1819	0.45	0.32	0.46	0.32	0.64				50S ribosomal protein L17
Sll1811		0.59							50S ribosomal protein L18
Sll1802		0.59							50S ribosomal protein L2
Srl1678			0.67	0.61					50S ribosomal protein L21
Sll1803	0.53	0.64	0.52	0.65					50S ribosomal protein L22
Sll1807		0.48							50S ribosomal protein L24
Ssr2799	0.00		0.58						50S ribosomal protein L27
Ssr1604	0.50	0.37	0.55						50S ribosomal protein L28
Sll1810		0.35		0.43	0.57				50S ribosomal protein L6
Sll1927				0.63					ABC transporter
Slr1694		0.63							Activat or photopigment and puc expression
Slr0083			0.55	0.39					ATP-dependent RNA helicase DeaD
Sll0834							0.42		Bicarbonate transporter
Sll1223		0.60							Bidirectional hydrogenase complex protein HoxU
Slr1839			0.66	0.58					Carbon dioxide concentrating mechanism protein CcmK
Sll1292								0.62	CheY family protein
Slr1641			0.64						ClpB protein
Sll1258					0.58				Deoxycytidine triphospahate deaminase
Sll1327			0.57	0.51					F0F1 ATP synthase subunit gamma
Sll0513						0.65			Franesyl-diphospahate farnesyltrasferas
Ssl0020							0.59		Ferredoxin
Sll0567					0.65				Ferric uptake regulation protein
Sll0248	0.00	0.00							Flavodoxin FldA
Sll1370					0.62				GDP-mannose pyrophosphorylase
Sll0207								0.59	Glucose-l-phosphate thymidylyltransferase
Slr0288					0.66	0.64			Glutamate--ammonia ligase
Sll1019	0.57	0.63							Glyoxalase II
Slr1123	0.62								Guanylate kinase
Sll0764					0.66				High-affinity branched- chain amino acid transport
Slr2099							0.57		Hybrid sensory kinase
Slr0546					0.61				indole-3-glycerol-phosphate synthase
Sll1214							0.66	0.63	Magnesium-protoprphyrin IX monomethyl ester cyclase
Sll0223								0.63	NAD(P)H-quinone oxidoreductase subunit 2
Slr0844								0.64	NAD(P)H-quinone oxidoreductase subunit F
Slr077			0.55						NifS protein
Slr2005				0.61					Periplasmic protein, function unknown
Sll0064					0.65				Putative polar amino acid transport system
Slr1410		0.61							Periplasmic WD-repeat protein
Slr1615		0.57							Perosamine synthetase
Sll0226	0.51		0.56						Photosystem I assembly protein Ycf4
Slr1894				0.60					Probable DNA-binding stress protein
Sll0772					0.57	0.57			Probable porin; major outer membrane protein
Slr1861				0.59					Probable sigma regulatory factor
Slr0677						0.64			Putative biopolymer transport protein exbB-like 2
Slr1531				0.66					Signal recognition particle protein
Slr1139							0.63	0.66	Thioredoxin
Sll0615								0.61	Transmembrane protein FT 27
Sll0518, Sll0596, Sll0783, Sll0872, Sll1118, Sll1304, Slr0007, Slr0147, Slr0168, Slr0723, Slr1260, Slr1363, Slr1385, Ssr2254									Hypothetical proteins

*Proteins with 1.5 fold change and *p- *value less than 0.05.

**Hypothetical proteins listed with gene ID only, full information in Additional file 1: Table S1.

***Blank representsthat ratio can’t be claculated for the condition.

### Membrane-bound proteins significantly induced by hexane

Pathway enrichment analysis showed that “ABC transporters” (KO02010) was differentially regulated by hexane exposure, among which four ABC transporters were matched to the KEGG pathway KO02010 at 48 h ( Additional file 2: Table S2). Further analysis, however, showed that more putative transporters were up-regulated by the hexane treatment (Table 1). Induction of transporters by stress or toxic solvent has been reported in many microbes, such as *acrAB-tolC* in *E. coli* and *srpABC* in *Pseudomonas putida* which have been shown to export hexane, heptane, octane, octanol and nonane, and the enhanced expression of these genes were related to high tolerance [48,49]. Up-regulation of transporters has been reported for various stresses in many other cyanobacterial species, such as arsenate resistance in *Anabaena variabilis*, salinity in *Synechocystis*[50-53]. In *Synechocystis* sp. PCC 6803, we recently found that five putative transporters involved in transporting of different substrates (*i.e.* polar amino acid, bicarbonate, iron, Na^+^/K^+^) were up-regulated by ethanol exposure [45]. Upon hexane exposure, a spectrum of putative transporters, including five transporters involved in bicarbonate transporting (Slr0041, Slr0043, Sll0834, Slr1512, Sll1734), two involved in cation transporting (Slr2131, Sll0672), two involved in Na^+^ and K^+^ transporting (Sll0689, Sll0493), one involved in mercuric transporting (Ssr2857), were up-regulated (Table 1). In *Synechocystis* sp. PCC 6803, the *slr004*0, *slr0041*, *slr0043*, and *slr0044* genes, forming an operon with a putative porin gene (*slr0042*), were induced under low CO_2_ stress conditions to increase the bicarbonate transporting [54]. Slr1512 was previously found essential to Na^+^-dependent HCO_3_^-^ transport and may play a crucial role in carbon acquisition when CO_2_ supply is limited [55]. *sll0493* encodes an NAD^+^-binding peripheral membrane protein (KtrA) that by working with a K^+^ transporters (KtrB; Slr1509) and KtrE (Slr1508), played a crucial role in the early phase of cell turgor regulation after hyperosmotic shock in *Synechocystis*[56]. Although the preliminary evidence suggested that these transporters may directly involve in hexane transporting, their up-regulation implicated the important roles for the hexane tolerance.

In addition, our proteomic analysis showed that many proteins located on cell membrane were up-regulated (Table 1), including a plasma membrane protein essential for thylakoid formation Sll0617 (*vipp1*) with a similar gene, *pspA*, in *E. coli* up-regulated by organic solvents [35], a periplasmic iron-binding protein (Slr0513) [57], four periplasmic proteins with unknown function (Slr1160, Slr2144, Slr1513, Sll0319), a salt-enhanced periplasmic protein (Sll1549). Meanwhile, several membrane-bound proteins were also found down-regulated by hexane exposure (Table 2). Although the exact function of these proteins was mostly unknown, the significant changes occurred at the cell membrane level may represent an important resistance strategy against hexane toxicity.

Bacteria use a variety of secretion systems to transport proteins beyond their cell membrane in order to interact with their environment. In *E. coli* and other gram-negative bacteria, one of the major translocation systems is the twin arginine translocation pathway consisted of TatA, TatB and TatC to export folded proteins across the cytoplasmic membrane [58]; and another is the Sec-dependent protein translocation system whose complex molecular machine comprises a flexible transmembrane conduit coupled to a motor-like component [59]. Our proteomics analysis showed that hexane treatment induced the expression of both systems in *Synechocystis* sp. PCC 6803. The putative TatA protein (Slr1046) of twin arginine translocation pathway and SecF (Slr0775) of the Sec-dependent protein translocation system were both up-regulated (Table 1).

### Regulatory systems regulated by hexane

Two-component system (TCS) is an important signal transduction mechanism employed by prokaryotes to survive the complex and volatile environments [60], and has been involved in various stress responses in cyanobacteria [61,62]. In our previous work, two response regulators (Slr1783, Slr1909) were found up-regulated by ethanol exposure; however, these two response regulators were not differentially regulated by hexane under our experimental conditions [45]; meanwhile, four different regulatory proteins were up-regulated, and two regulatory proteins were down-regulated by hexane exposure (Table 1, 2). Interestingly, up-regulated Slr1697 of a serine/threonine kinase and up-regulated Sll0043 of a positive phototaxis hybrid histidine kinase (homologous to chemotaxis protein CheA) were both required for cell motility and chemotaxis [63,64]. Chemotaxis and flagellar motility are essential mechanisms by which bacteria use to adapt to and survive in environments suffered with various natural stresses [65], our results showed that the *Synechocystis* cells may adapt the similar mechanism in dealing with the stress caused by hexane. In addition, the fact that a different set of regulatory proteins were involved in hexane stress from those in ethanol stress implicated that variable resistance mechanisms were used by the cells for the biofuel products of different chemical properties [45].

#### Common stress response

Early studies have defined a set of common stress responses that cells will typically initiate upon stresses from organic solvent or biofuel products, including down-regulation of ribosomal proteins, induction of heat shock proteins to aid with proper protein folding, induction of oxidative stress response, and modification of cell membrane [66-68]. Our proteomic analysis showed that the initiation of these common stress response programs were also seen in the hexane-treated *Synechocystis* cells, although the protein species used for each of the function could vary between microbial species (Table 1, 2). For example, a heat shock protein (GrpE, Sll0057) was induced by hexane in the *Synechocystis* cells, while in *E. coli* treated with one of the hydrophobic organic solvents, *n*-hexane or cyclooctane, a 28 kDa phage-shock protein PspA was induced [69]. While in the ethanol-treated *Synechocystis* cells, two heat shock proteins, GroES (Slr2075) and GrpE (Sll0057) were up-regulated [45]. Oxidative stress responses have been reported for cells under treatment of organic solvents as they induced production of highly reactive oxygen species (ROS). Without exception, ROS formation has been suggested playing a important role in *n*-hexane induced alterations in cell proliferation and membrane integrity [70]. In addition to two responsive glutathione peroxidase discussed above, our proteomic analysis showed that a bacterioferritin comigratory protein (Slr0242), a rehydrin (Slr1198) and a methionine sulfoxide reductase A (Sll1394) were also induced in response to the oxidative stress caused by hexane in *Synechocystis* (Table 1). Studies of solvent-tolerant microbes found that cells can modify fatty acid composition or other accessory molecules of their cell membrane to block the entry of solvents [66,67]. Up-regulation of Slr1761, a peptidylprolyl *cistrans* isomerase which catalyses the *cistrans* isomerization of proline imidic peptide bonds in oligopeptides in both prokaryotic and eukaryotic cell envelope upon stress, was found in hexane-treated cells, similar up-regulation of Slr1761 by ethanol was also observed previously *Synechocystis*[45].

## Conclusions

Alkane compounds of varying carbon lengths have been proposed as a good alternative to gasoline. However, one of the major hurdles needed to be overcome is that alkanes typically exhibit toxicity to microbes, especially cyanobacterial cells. Towards this end, we employed a global-based quantitative proteomics approach with iTRAQ - LC-MS/MS technologies to reveal the responses of *Synechocystis* to hexane, a representative of alkane. Using a cutoff of 1.5-fold change and a *p*-value less than 0.05, a total of 164 up-regulated and 77 down-regulated proteins were determined. Function annotation and KEGG pathway enrichment analyses showed that common stress responses which have been reported for other microbes under organic solvent/biofuel stress were also induced in *Synechocystis*[66,67]. Notably, the analysis revealed the induction of a large number of transporters and membrane-bound proteins, proteins related to sulfur relay system, oxidative stress and photosynthesis, suggesting that they were among the major protection mechanisms against hexane. In this study hexane is added exogenously, however, it is expected that the general responses uncovered by the study will be similar to that caused by hexane generated intracellularly, although intracellular hexane may be more toxic at lower concentration. Nevertheless, the study provided the first comprehensive view of the complicated molecular mechanism employed by cyanobacterial model species, *Synechocystis* to defend against hexane stress. Moreover, the proteomic analysis identified a list of potential target genes/proteins which can be engineered to generate stress-resistant hosts for high efficiency production of hexane [71,72].

## Methods

### Bacterial growth condition and hexane treatment

*Synechocystis* sp. PCC 6803 was obtained from American Type Culture Collection (ATCC) and grown in BG11 medium (pH 7.5) under a light intensity of approximately 50 μmol photons m^-2^ s^-1^ in an illuminating incubator operated at 130 rpm and at 30°C (HNY-211B Illuminating Shaker, Honour, China) [73-75]. Cell density was measured on a UV-1750 spectrophotometer (Shimadzu, Japan). For growth and hexane treatment, the 10 mL fresh cells of OD_730_ 0.5 were collected by centrifugation at 8000 x *g* for 10 min at 4°C, and then were inoculated into 50 mL BG11 liquid medium in a 250-mL flask. Hexane of varying concentration was added at the beginning of cultivation. 1 mL of culture samples were collected and measured with a spectrophotometer at OD_730_ every 12 h. Morphology of *Synechocystis* under control and treatment conditions was observed using a BX43 fluorescence microscope (Olympus, Japan). Growth experiments were repeated at least three times to confirm the growth patterns. Cells for proteomics analysis were collected by centrifugation at 8,000 x *g* for 10 min at 4°C.

### Protein preparation and digestion

For each sample, 10 mg of cells were frozen by liquid nitrogen immediately after centrifugation and washed with phosphate buffer (pH 7.2). The cells were broken with sonication cracking at low temperature. Cell pellets were then resuspended in a lysis buffer (8 M urea, 4% CHAPS, 40 mM Tris–HCl), with 1 mM PMSF and 2 mM Ethylenediaminetetraacetic acid (EDTA) (final concentration). After 5 min of vigorously vortex, dithiothreitol (DTT) was also added to a final concentration of 10 mM. After mix, the sample were centrifuged for 20 min at 20,000 x *g*, and the supernatant was mixed well with ice-cold acetone (1:4, *v*/*v*) with 30 mM DTT. After repeating this step twice, supernatants were combined and precipitated at −20°C overnight, and stored at −80°C prior to sample if not for immediate use. For digestion, protein pellet from previous step was resuspended in digestion buffer (100 mM triethylammonium bicarbonate TEAB, 0.05% *w***/***v* sodium dodecyl sulfate, SDS) to a final concentration of 1 mg/mL (total protein measured by bicinchonic acid assay (Sigma, St. Louis, MO)). Equal aliquots (500 μg) from each lysate were then digested with trypsin overnight at 37°C (Sigma; 1:40 *w*/*w* added at 0 and 2 h) and lyophilized.

### iTRAQ labeling

The iTRAQ labeling of peptide samples derived from control and hexane treatment conditions were performed using iTRAQ reagent Multiplex kit (Applied Biosystems, Foster City, CA) according to manufacturer’s protocol. For each time point (*i.e.* 24 h or 48 h), four samples (two biological replicates for control and two biological replicates for hexane-treated samples) were iTRAQ labeled. The peptides labeled with respective isobaric tags, incubated for 2 h and vacuum centrifuged to dryness. The labeled control and hexane treatment replicate samples were 1:1 pooled, and generating four combinations of samples for each time point, which were reconstituted in Buffer A (10 mM KH_2_PO_4_, 25% acetonitrile, pH 2.85). The iTRAQ labeled peptides were fractionated using PolySULFOETHYL ATM SCX column (200 x 4.6 mm, 5 μm particle size, 200 A° pore size) by HPLC system (Shimadzu, Japan) at flow rate 1.0 ml min^-1^. The 50 min HPLC gradient consisted of 100% buffer A (10 mM KH_2_PO_4_, 25% acetonitrile, pH 2.85) for 5 min; 0-20% buffer B (10 mM KH_2_PO_4_, 25% ACN, 500 mM KCL, pH 3.0) for 15 min; 20-40% buffer B for 10 min; 40-100% buffer B for 5 min followed by 100% buffer A for 10 min. The chromatograms were recorded at 218 nm. The collected fractions were desalted with Sep-Pak® Vac C18 cartridges (Waters, Milford, Massachusetts), concentrated to dryness using vacuum centrifuge and reconstituted in 0.1% formic acid for LC-MS/MS analysis.

### LC-MS/MS proteomic analysis

The mass spectroscopy analysis was performed using a AB SCIEX TripleTOF™ 5600 mass spectrometer (AB SCIEX, Framingham, MA, USA), coupled with online micro flow HPLC system (Shimadzu, JAPAN) as described before [76,77]. The peptides were separated using nanobored C18 column with a picofrit nanospray tip (75 μm ID x 15 cm, 5 μm particles) (New Objectives, Wubrun, MA). The separation was performed at a constant flow rate of 20 μl min^-1^, with a splitter to get an effective flow rate of 0.2 μl min^-1^. The mass spectrometer data acquired in the positive ion mode, with a selected mass range of 300–2000 m/z. Peptides with +2 to +4 charge states were selected for MS/MS. The three most abundant peptides above a 5 count threshold were selected for MS/MS and dynamically excluded for 30 s with ±30 mDa mass tolerance. Smart information-dependent acquisition (IDA) was activated with automatic collision energy and automatic MS/MS accumulation. The fragment intensity multiplier was set to 20 and maximum accumulation time was 2 s. The peak areas of the iTRAQ reporter ions reflect the relative abundance of the proteins in the samples.

### Proteomic data analysis

The data acquisition was performed with Analyst QS 2.0 software (Applied Biosystems/MDS SCIEX). Protein identification and quantification were performed using Mascot 2.3.02 (Matrix Science, London, United Kingdom) [78]. For iTRAQ quantification, the peptide for quantification was automatically selected by the algorithm to calculate the reporter peak area, error factor (EF) and *p*-value. The resulting data set was auto bias-corrected to get rid of any variations imparted due to the unequal mixing during combining different labeled samples. Genome sequence and annotation information of *Synechocystis* were downloaded from NCBI and the Comprehensive Microbial Resource (CMR) of TIGR (http://www.tigr.org/CMR) (April 22, 2012) [38]. Proteins with 1.5 fold change between hexane-treated and control samples and *p*-value of statistical evaluation less than 0.05 were determined as differentially abundant proteins. Metabolic pathway enrichment analysis of the responsive proteins was conducted according to the information from the KEGG Pathway Database using the following formula:

(1) $$P=1\sum_{i=o}^{m-1}\frac{\left(\begin{array}{c} M\\ i \end{array}\right)\left(\begin{array}{c} N-M\\ n-i \end{array}\right)}{\left(\begin{array}{c} N\\ n \end{array}\right)}$$

where *N* is the number of all proteins with KEGG pathway annotation information, *n* is the number of the differentially regulated proteins with KEGG pathway annotation information, *M* is the number of proteins with a given KEGG pathway annotation, *m* is the number of the differentially regulated proteins with a given KEGG pathway annotation. The KEGG pathways with *p*-value less than 0.05 were considered as enriched KEGG pathways by the hexane responsive proteins.

## Abbreviations

iTRAQ: Isobaric tag for relative and absolute quantitation; LC-MS/MS: Liquid chromatography-tandem mass spectrometry; KEGG: Kyoto Encyclopedia of Genes and Genomes; TCS: Two-component system.

## Competing interests

The authors declare that they have no competing interests.

## Authors’ contributions

LC, JW, and WZ conceived of the study. LC, LW, JL, JQ and WZ drafted the manuscript. JL, LC, JW, and WZ carried out cultivation, protein isolation and proteomics analysis. LC, JW and WZ finish the statistical analysis for proteomics data. “All authors read and approved the final manuscript.”

## Supplementary Material

Additional file 1 **Table S1. **Full list of identified proteins and their ratio across conditions *. Click here for file

Additional file 2 **Table S2. **KEGG pathway enrichment analysis of the differentially regulated proteins *. Click here for file
